# Non‐detached hamstring tendon anterior cruciate ligament reconstruction demonstrates comparable outcomes to the traditional detached hamstring tendon technique: A systematic review and meta‐analysis

**DOI:** 10.1002/jeo2.70705

**Published:** 2026-05-05

**Authors:** Khaled Skaik, Helena Son, Benjamin Blackman, Marc Daniel Bouchard, Prushoth Vivekanantha, Amit Meena, Shahbaz S. Malik, Darren de SA

**Affiliations:** ^1^ Faculty of Medicine and Health Sciences McGill University Montreal Quebec Canada; ^2^ Michael G. DeGroote School of Medicine McMaster University Hamilton Ontario Canada; ^3^ University of Limerick School of Medicine Limerick Ireland; ^4^ Division of Orthopaedic Surgery McMaster University Hamilton Ontario Canada; ^5^ KNEECARES—The Superspeciality Knee Clinic Jaipur India; ^6^ Division of Orthopaedic Surgery Queen's University Kingston Ontario Canada; ^7^ Birmingham Knee School Birmingham UK; ^8^ Department of Orthopaedics Worcestershire Acute Hospitals NHS Trust Worcester UK

**Keywords:** anterior cruciate ligament reconstruction, detached hamstring tendon, IKDC, non‐detached hamstring tendon, preserved hamstring tendon

## Abstract

**Purpose:**

To compare clinical and radiological outcomes of anterior cruciate ligament reconstruction (ACLR) using non‐detached hamstring tendon (NDHT) versus conventional detached hamstring tendon (DHT) technique.

**Methods:**

Embase, PubMed, Ovid Medline and CINAHL databases were searched from inception to October 2025. Inclusion criteria included comparative studies of clinical or radiographic outcomes between NDHT and DHT. Studies were excluded if they were non‐comparative or if they involved extra‐articular procedures. The primary outcome was the post‐operative International Knee Documentation Committee (IKDC) score. Secondary outcomes included Lysholm, Tegner, KT‐1000 (knee testing 1000 [laxity arthrometer]) scores, rate of return‐to‐sport (RTS), retear rate and graft maturation. Data were pooled using random‐effects models with significance set at *p* < 0.05. Sensitivity analyses included randomized trials only.

**Results:**

A total of 11 studies (*n* = 731; 354 NDHT, 377 DHT patients) met inclusion criteria. IKDC scores at ≥12 months (mean difference [MD] = 0.67, 95% confidence interval [CI]: –2.67 to 3.94, *p* = 0.69), Lysholm scores at ≥6 months (MD = –1.02, 95% CI: –2.52 to 0.49, *p* = 0.19), Tegner scores at ≥6 months (MD = 0.18, 95% CI: –0.19 to 0.55, *p* = 0.34), KT‐1000 arthrometer at ≥24 months (MD = –0.29, 95% CI: –0.67 to 0.30, *p* = 0.14), RTS rates at ≥12 months (risk ratio [RR] = 1.00, 95% CI: 0.94 to 1.07, *p* = 0.89) and retear rates at ≥12 months (RR = 0.63, 95% CI: 0.17 to 2.40, *p* = 0.50). None of these findings changed in sensitivity analysis. The NDHT demonstrated significantly better graft maturation at 6 and 12 months (*p* < 0.05), but these differences disappeared by 24 months.

**Conclusions:**

Preserving the tibial insertion of the hamstring graft in ACLR (NDHT) resulted in comparable clinical outcomes to those of the detached technique (DHT), with no significant differences observed between the two approaches. Future studies should assess potential cost and time benefits of NDHT.

**Level of Evidence:**

Level IV.

**Abbreviations**
ACL: anterior cruciate ligament
ACLR: anterior cruciate ligament reconstruction
CI: confidence interval
DHT: detached hamstring tendon
IKDC: International Knee Documentation Committee
KT‐1000: knee testing 1000 (laxity arthrometer)
MD: mean difference
MRI: magnetic resonance imaging
NDHT: non‐detached hamstring tendon
PRISMA: Preferred Reporting Items for Systematic Reviews and Meta‐analyses
RCT: randomized controlled trial
ROB2: risk of bias tool version 2
ROBINS‐I: Risk of Bias In Non‐randomized Studies—of Interventions
RR: risk ratio
RTS: return to sport
SD: standard deviation
SNQ: signal‐to‐noise quotient
ST: semitendinosus
TTW: tibial tunnel widening

## INTRODUCTION

Anterior cruciate ligament (ACL) tears are among the most frequently encountered knee injuries [5, 17, 25, 35]. A range of graft options have been utilized in ACL reconstruction (ACLR) surgeries to restore knee stability, including hamstring, bone–patellar tendon–bone and quadricep tendon grafts. Hamstring tendon techniques are particularly common due to their good strength, low donor site morbidity, minimal anterior knee pain and excellent long‐term outcomes [9, 26, 31, 34, 35]. In fact, hamstring tendon remains the predominant graft in 80.3% of the surveyed population of orthopaedic surgeons [11, 44]. The traditional method, known as detached hamstring graft (DHT), involves harvesting the gracilis (G) and semitendinosus (ST) tendons by detaching them from their tibial insertion [18, 21, 41, 55]. Detaching the hamstring tendons has also become a widely adopted strategy to achieve larger graft diameters (≥ 8 mm) of various multi‐strand configurations aimed to reduce the risk of graft failure and revision [43].

Following ACLR, all grafts undergo a biological remodelling process composed of three key phases: necrosis, proliferation and ligamentization [8, 30]. Notably, the necrosis phase is associated with compromised biomechanical strength, which can delay or even prevent patients from returning to regular activities and sports [4, 16, 24, 30, 33, 45].

To address this limitation, a range of biological and biomechanical augmentation strategies have been explored. These include biological augmentation [23], biomechanical support using suture tape or internal brace constructs to protect the graft during early healing [19, 48], remnant‐preserving techniques aimed at retaining native mechanoreceptors and vascularity [49] and the use of post‐operative biologics such as platelet‐rich plasma (PRP) to enhance healing potential [36].

In addition, there has been growing interest in preserving the distal insertion of the ST tendon at the pes anserinus, detaching it only at its musculotendinous junction proximally to maintain vascularity from the tibial attachment [24]. In this approach, known as the non‐detached hamstring graft (NDHT), retaining the tibial insertions of both the G and ST tendons has been shown to enhance construct stability through double tibial fixation, facilitate superior graft remodelling and preserve vascular and neural supply for proprioception [6, 18, 22, 28, 29, 52, 54].

The objectives of the present study were to determine whether preserving the hamstring tibial insertion leads to superior clinical and radiological outcomes compared to detaching the hamstring tendon. Specifically, this study aimed to (1) assess clinical differences between DHT and NDHT groups, (2) compare rates of re‐tears and rate of return‐to‐sport (RTS) and (3) compare graft maturation between both groups. It was hypothesized that NDHT and DHT would have comparable clinical outcomes at all follow‐up periods.

## METHODS

### Study design

This review followed the Cochrane Handbook for Systematic Reviews of Interventions PRISMA (Preferred Reporting Items for Systematic Reviews and Meta‐analyses) guidelines [27]. As this study involved secondary analysis of published data, institutional review board (IRB) approval was not required. A comprehensive search was performed across multiple electronic databases, including CINAHL, Embase, PubMed and Ovid Medline, covering studies up to 28 October 2025. Keywords such as ‘anterior cruciate ligament reconstruction’, ‘ACL surgery’, ‘pedicle preservation’, ‘pedicled hamstring’ and ‘detached graft’ were combined using Boolean operators ‘AND’ and ‘OR’. The full search strategy is available in Supporting Information S1.

### Eligibility criteria

Studies eligible for inclusion involved comparative studies. This included randomized controlled trials (RCTs), prospective or retrospective cohort studies and case‐control studies investigating patients undergoing primary ACLR using a hamstring tendon graft. The intervention of interest was the preservation of at least one hamstring tendon's tibial insertion during ACLR, while the comparator is the standard technique involving detachment of the hamstring tendon's tibial insertion. Included studies reported at least one of the following outcomes: clinical outcomes (such as IKDC, Lysholm, Tegner scores or RTS) or graft maturation or remodelling as assessed by magnetic resonance imaging (MRI) (including measures such as signal/noise ratio and tunnel enlargement).

The exclusion criteria included revision ACLR, use of non‐hamstring grafts such as patellar tendon and quadriceps tendon, animal studies, cadaveric, biomechanical (non‐clinical) studies or non‐comparative studies. Any study that incorporated extra‐articular procedures, such as extra‐articular or lateral plasty, in addition to the ACLR was excluded. Studies lacking extractable data for relevant clinical or radiological outcomes or those not reporting at least one predefined outcome of interest were also removed. Furthermore, studies in which tibial insertion preservation was combined with other remnant‐preservation or augmentation techniques were excluded unless tibial‐insertion–specific data could be extracted separately.

### Study selection

All search results were imported into Covidence systematic review software (Veritas Health Innovation) for deduplication and blinded screening. Two reviewers (K. S. and H. S.) independently screened titles and abstracts, followed by full‐text reviews to determine final eligibility. Disagreements at either stage were resolved through discussion or adjudication by a third reviewer (M. D. B.). Inter‐reviewer agreement during screening was assessed using the kappa (*κ*) statistic. Agreement levels were predefined as follows: *κ* = 0.91–0.99, almost perfect; *κ* = 0.71–0.90, substantial; *κ* = 0.61–0.70, high; *κ* = 0.41–0.60, moderate; *κ* = 0.21–0.40, fair and *κ* ≤ 0.20, no agreement [39].

### Primary and secondary outcomes

Primary outcome for this study was the post‐operative International Knee Documentation Committee (IKDC). Secondary outcomes included the post‐operative (1) Lysholm score, (2) Tegner score, (3) KT‐1000 (knee testing 1000 [laxity arthrometer]) laxity arthrometer measurement scores, (4) rate of RTS and retear between both groups and (5) graft maturation using signal‐to‐noise quotient (SNQ) or other measures.

### Risk of bias assessment

For randomized studies, the Cochrane risk of bias tool version 2 (ROB2) was used for the RCTs to assess the quality of the trial for selection, attrition, detection and reporting biases [14]. Based on this assessment, each domain was assigned an overall risk of bias: low, unclear or high. The Risk of Bias In Non‐randomized Studies—of Interventions (ROBINS‐I) tool was used for the non‐randomized studies [14, 42]. This tool evaluates the risk of bias across several areas: confounding, participant selection, intervention classification, deviations from intended interventions, missing data, outcome measurement and selection of reported results [42].

### Statistical analysis

Meta‐analyses were performed using a random effects model (DataParty, Python 3.8.10). Forest plots were generated for six outcomes: Lysholm score, KT‐1000 arthrometer measurements, Tegner activity scale, RTS, re‐tear rate and IKDC score at the latest follow‐up of minimum 6 months. Sensitivity analysis was conducted for all outcomes using only RCTs. Continuous outcomes were pooled using mean differences (MDs) with 95% confidence intervals (CIs), while dichotomous outcomes were analysed using risk ratios (RRs). If a study did not report standard deviation, it was estimated using the formula ‘standard deviation (SD) = range/4’ [15] as applied in two studies [33, 50]. Forest plots were created using DataParty (Python 3.8.10). The *I* ^2^ statistic was used to assess heterogeneity, with values between 25%–49% considered low, 50%–74% moderate and ≥75% high heterogeneity. Statistical significance was defined as a two‐tailed *p* value of less than 0.05. Outcomes that could not be meta‐analysed due to limited data or inconsistent reporting were summarized descriptively.

## RESULTS

### Study characteristics and patient population

**Figure 1:**
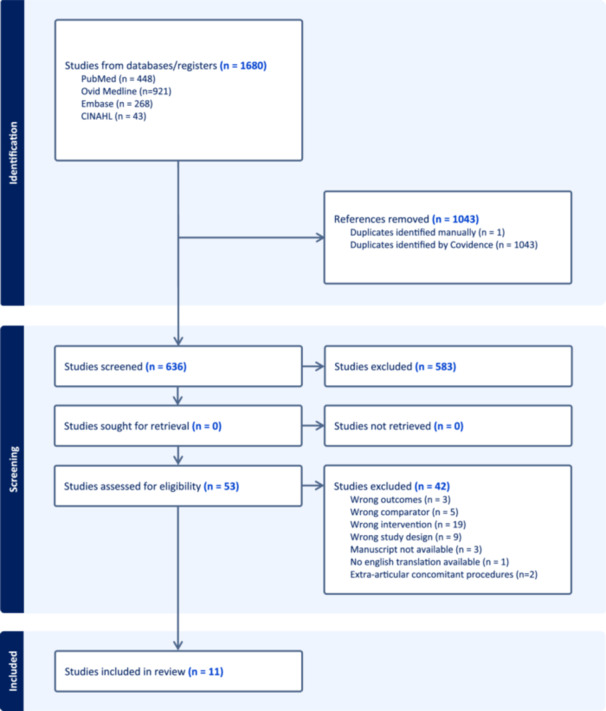
Preferred reporting items for systematic reviews and meta‐analyses flow diagram representing a systematic review on detached versus preserved hamstring tibial insertion tendon on anterior cruciate ligament reconstruction surgery.

The initial search identified 1680 studies, and 1043 duplicates were excluded. Of the remaining 636 studies, 583 were excluded based on title and abstract screening. After full‐text review and eligibility assessment, 11 studies met the inclusion criteria (Figure 1). Other studies were excluded due to reporting the wrong outcomes, having a wrong comparator, wrong intervention or combining ACLR with extra‐articular procedures [12, 54] (Figure 1). Of the included studies, six were RCTs [3, 10, 13, 20, 33, 56], three were prospective [29, 37, 47] and two were retrospective cohort studies [40, 50]. There was considerable agreement during both title and abstract screening *κ* = 0.74 [95% CI 0.63, 0.85] and during full text review *κ* = 0.87 [95% CI 0.72, 1.00].

A total of 731 patients (731 knees) were included, of which 354 with DHT and 377 with NDHT were included in this study (Table 1). The age of patients ranged from 18.0 to 49.0 years, with the proportion of male patients spanning from 57.8% to 96.9% across the studies. The follow‐up periods ranged from 3 to 60 months across included studies (Table 1).

**Table 1:** Study characteristics and patient population of included studies.

Primary author et al. (year) [Reference]	Study design	Level of evidence	Number of patients (*n*)	Number of knees (*n*)	Knees with detached hamstring (DHT) (*n*)	Knees with non‐detached hamstring (NDHT) (*n*)	Percentage male (%)	Mean age, years (SD) [range]	Type and configuration of hamstring graft	Graft diameter, mm Mean (SD)	Follow‐up, months
Baldassarri et al. (2019) [3]	Prospective RCT	I	59	59	28	31	DHT: 82.1%	24.9 ± 5.4 [17.5–31.7]	DHT: Quadrupled ST tendon	DHT: NR	6, 12, 24, 36 and 48 months
							NDHT: 74.2%	DHT: 24.7	NDHT: Doubled hamstring tendons, single‐bundle reconstruction. ST and G tibial insertion preserved.	NDHT: NR	
								NDHT: 25.2			
Espinoza Dumlao III et al. (2020) [10]	Prospective RCT	I	32	32	18	14	DHT: 94.4%	DHT: 23.2 [18.0–40.0]	DHT: Detached four‐strand HT graft.	DHT: NR	0.5, 1, 2, 3, 4, 5, 6 and 7 months
							NDHT: 78.6%	NDHT: 22.9 [20.0–34.0]	NDHT: Four‐strand HT graft with preserved tibial attachment.	NDHT: NR	
Gupta et al. (2017) [13]	Prospective RCT	I	110	110	55	55	DHT: 94.5%	DHT: 27.2 ± 5.7	DHT: ST and G quadrupled graft	DHT: NR	3, 6, 12 and 24 months
							NDHT: 96.4%	NDHT: 27.0 ± 7.5	NDHT: ST and G quadrupled graft, both preserved.	NDHT: NR	
Liu et al. (2018) [20]	Prospective RCT	I	37	37	19	18	DHT: 63.2%	DHT: 29.4 ± 5.3	DHT: Four‐strand double‐looped hamstring autograft	DHT: NR	3, 6, 12 and 24 months
							NDHT: 72.2%	NDHT: 31.5 ± 6.6	NDHT: Four‐strand double‐looped hamstring autograft. ST and G tibial insertion preserved.	NDHT: NR	
Papachristou et al. (2008) [29]	Prospective cohort	II	41	41	23	18	DHT: 88.9%	DHT: 25.8 [18.0–38.0]	DHT: Double‐bundle, double tibial tunnel, both bundles detached	DHT: NR	DHT: 16–30 months
							NDHT: 91.3%	NDHT: 23.7 [18.0–29.0]	NDHT: Double‐bundle, double tibial tunnel. Only G tibial insertion is preserved	NDHT: NR	NDHT: 12–24 months
Ruffilli et al. (2016) [33]	Prospective RCT	I	40	40	20	20	80.0%	27.5 [18.0–49.0]	DHT: Quadruple‐stranded ST tendon graft	DHT: 9.0 (SD NR)	3, 6, 12 and 24 months
							DHT: NR	DHT: NR			
							NDHT: NR	NDHT: NR	NDHT: Doubled ST and G tendon graft, both tibial insertions preserved.	NDHT: 9.0 (SD NR)	
Singh et al. (2022) [37]	Prospective cohort	II	52	52	26	26	DHT: 73.1%	DHT: 29.5 ± 9.8	DHT: Doubled ST‐gracilis graft	DHT: 8.1 (0.7)	1.5, 3 and 6 months
							NDHT: 69.2%	NDHT: 30.7 ± 10.0	NDHT: Doubled ST‐G graft, both tibial insertions preserved.	NDHT: 8.0 (0.7)	
Soni et al. (2021) [40]	Retrospective cohort	III	65	65	32	33	DHT: 96.9%	DHT: 23.2 ± 7.5	DHT: Free ST‐G graft. Configuration not specified	DHT: NR	≥24 months
							NDHT: 81.8%	NDHT: 23.0 ± 5.7	NDHT: ST and G tibial insertion preserved. Configuration not specified.	NDHT: NR	
Vari et al. (2023) [47]	Prospective cohort	II	180	180	90	90	DHT: 57.8%	DHT: 27.2 ± 9.4	DHT: Standard free quadruple‐stranded ST graft with detached tibial insertion	DHT: 8.6 (0.9)	12 months
							NDHT: 66.6%	NDHT: 27.7 ± 8.9	NDHT: Quadruple‐stranded ST graft. Only ST tibial insertion was preserved.	NDHT: 9.0 (0.7)	
Yilmaz (2023) [50]	Retrospective cohort	III	78	78	24	Six‐strand NDHT: 31	DHT: 67.0%	DHT: 31.0 [18.0–54.0]	DHT: Quadruple‐stranded HT graft without protecting the tibial insertion.	DHT: 8.0 [7.0–9.0]	1, 1.5, 2, 3 and 6 months
						Four‐thread NDHT: 23	Six‐strand NDHT: 13.0%	Six‐strand NDHT: 29.0 [19.0‐40.0]	Six‐strand NDHT: Six‐strand HT graft without severing the tibial insertion.	Six‐strand NDHT: 9.0 [8.0–10.0]	
							Four‐thread NDHT: 35.0%	Four‐thread NDHT: 32.0 [16.0–49.0]	Four‐thread NDHT: Quadruple‐strand HT graft without severing the tibial insertion.	Four‐thread NDHT: 8.0 [7.0–9.0]	
Zhang et al. (2020) [56]	Prospective RCT	I	37	37	19	18	DHT: 63.2%	DHT: 29.4 ± 5.3	DHT: Single‐bundle free hamstring tendon	DHT: NR	6, 12, 24 and 60 months
							NDHT: 72.2%	NDHT: 31.5 ± 6.6	NDHT: Single‐bundle hamstring tendon. ST and G tibial insertion preserved.	NDHT: NR	

Abbreviations: DHT, detached hamstring tendon graft; G, gracilis; HT, hamstring tendon; NDHT, non‐detached hamstring tendon graft; NR, not reported; RCT, randomized controlled trial; SD, standard deviation; ST, semitendinosus.

### Surgical technique of NDHT

All included studies preserved the tibial insertion of both the ST and G tendons, except for one study that preserved the ST only [47], and another study that preserved the G only [29] (Table 1). In the NDHT group, 10 studies employed a standard anatomic tunnel technique [3, 10, 13, 20, 33, 37, 40, 47, 50, 56], except one study used a double tunnel technique [29].

Double fixation was used in six studies [10, 20, 37, 47, 50, 56], while the remaining studies did not employ double fixation. Double fixation means the graft is secured on the tibial side by both hardware and the native tibial insertion, providing a ‘backup’ fixation [20, 56]. All studies used an EndoButton or a suture‐button device for femoral fixation and an interference screw for tibial fixation. The graft diameters in DHT and NDHT groups are summarized in Table 1.

### Additional procedures

Two studies reported additional meniscal procedures performed during ACL reconstruction in both DHT and NDHT groups [20, 33]. In one study, the NDHT group had 50% of cases undergoing partial meniscectomies and 22.2% undergoing meniscal repairs, whereas in the DHT group, 63.2% underwent partial meniscectomies and 21.1% underwent meniscal repairs [20]. Another study reported 70% of NDHT cases involved partial meniscectomies compared to 30% in the DHT group [33].

### Risk of bias assessment

**Figure 2:**
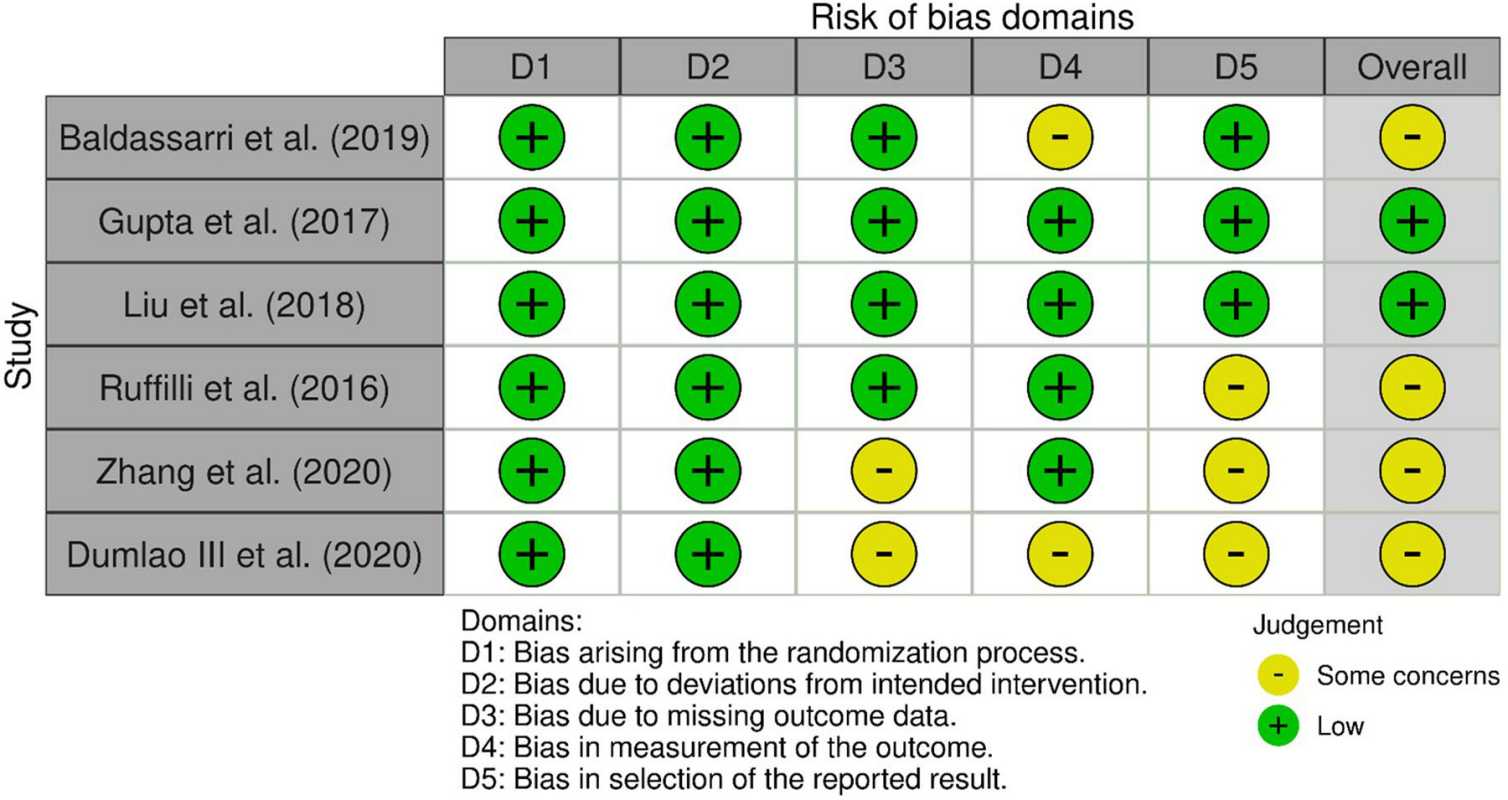
Risk of bias assessment using ROB2 tool for randomized controlled trials. ROB2, risk of bias tool version 2.

**Figure 3:**
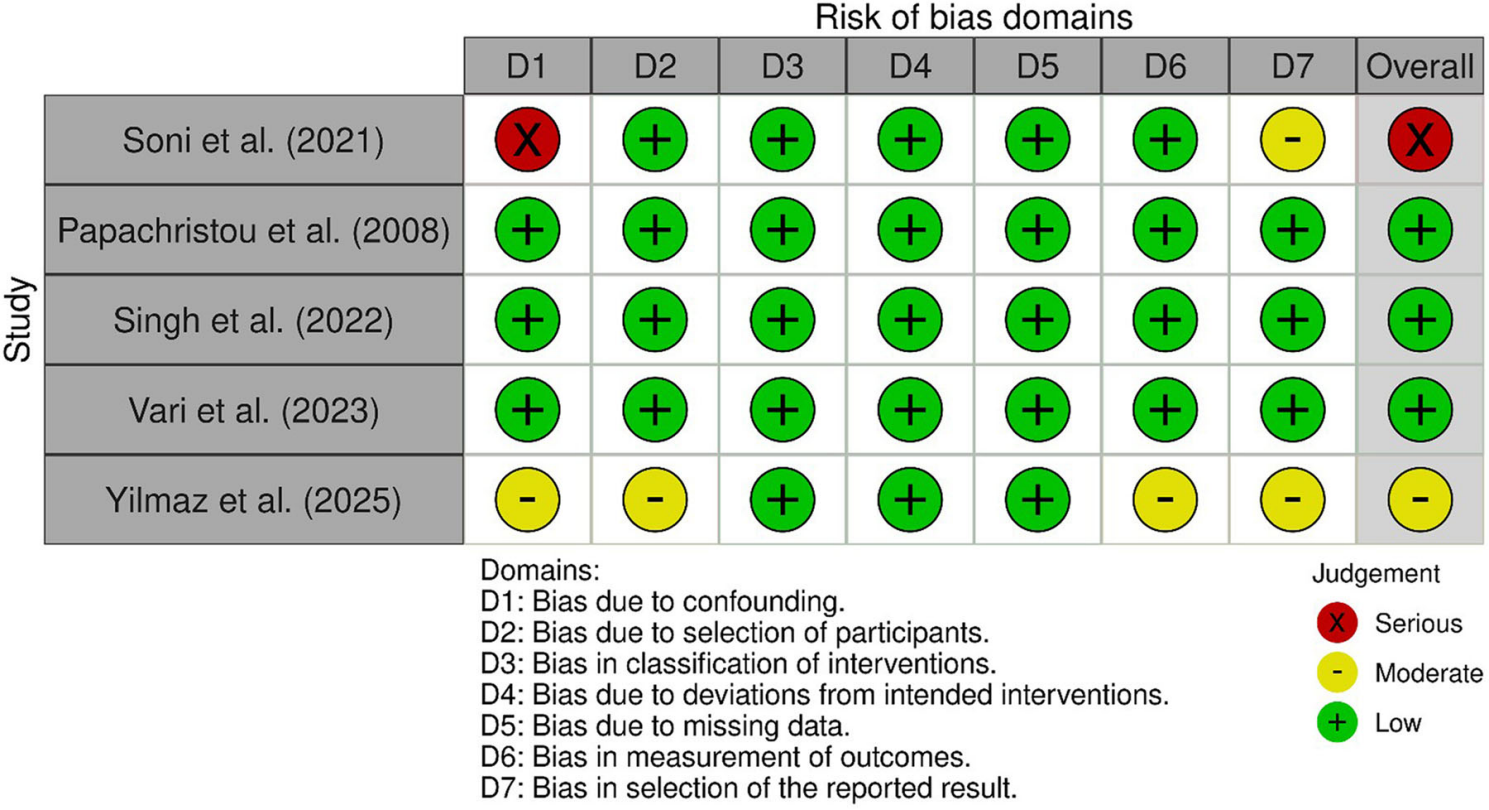
ROBINS‐I tool for non‐randomized included studies. ROBINS‐I, Risk of Bias In Non‐randomized Studies—of Interventions.

For RCTs, three studies had some risk of bias [3, 10, 33, 56], and two studies had low risk of bias [13, 20] (Figure 2). For non‐RCTs, three studies had low risk of bias [29, 37, 47], one had moderate risk [50] and one had serious risk [40] (Figure 3).

#### IKDC

A total of nine studies reported on IKDC scores at follow‐up periods of 3, 6, 12, 24 and 60 months [3, 10, 20, 29, 33, 47, 50, 56]. At 3 months, two studies found no significant difference in IKDC scores between DHT and NDHT groups [20, 33], but one study found a significant difference in IKDC (DHT: 54.61 ± 12.09 and NDHT: 70.38 ± 15.66, *p* = 0.0047), favouring the NDHT group [10]. At 6 months, four studies also reported no statistically significant difference between the groups [20, 33, 50, 56], except one study that reported significant difference (DHT: 68.07 ± 12.97 and NDHT: 82.77 ± 8.11, *p* = 0.0016), favouring the NDHT group [10].

At 12‐month follow‐up, a total of six studies reported on IKDC [20, 29, 33, 47, 56], of which all reported no statistical difference between DHT and NDHT except one study [3], which reported a significantly higher IKDC score (89.6) in the NDHT group compared to the DHT group (84.2), *p* < 0.01 [3, 20, 29, 33, 47, 56].

**Figure 4:**
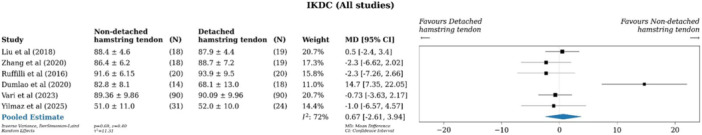
Meta‐analysis for studies reporting IKDC at the latest follow‐up. CI, confidence interval; IKDC, International Knee Documentation Committee; MD, mean difference.

On meta‐analysis across included studies at the latest follow‐up of minimum of 6 months, there was no significant difference on meta‐analysis (MD = 0.67 [95% CI: −2.67 to 3.94], *p* = 0.69) (Figure 4). These findings did not change on sensitivity analysis (MD = 1.91 [95% Cl: −3.55 to 7.36], *p* = 0.49) (Supporting Information S1: Figure 1).

#### Lysholm score

**Figure 5:**
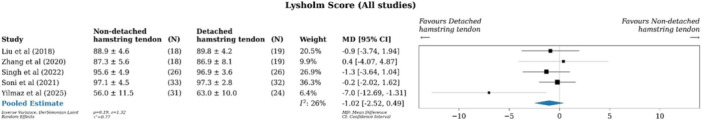
Meta‐analysis for studies reporting Lysholm score at the latest follow‐up. CI, confidence interval; MD, mean difference.

A total of five studies reported Lysholm scores at various follow‐up periods [20, 37, 40, 50, 56]. At 3‐month follow‐up, two studies reported no significant differences in Lysholm scores between the DHT and NDHT groups [20, 37]. At 6 months, three studies also found no significant differences between groups [20, 37, 50, 56]. At 12 months, three studies assessed Lysholm scores [20, 47, 56]. While two studies reported no significant differences, another study found a significantly higher median Lysholm score in the NDHT group (99; interquartile range [IQR], 95–100) compared to the DHT group (95; IQR, 91–99), *p* = 0.004 [47]. The meta‐analysis at the latest follow‐up of a minimum of 6 months showed no significant difference between the NDHT and DHT (MD = −1.02 [95% CI: −2.52 to 0.49], *p* = 0.19) (Figure 5). These findings did not change on sensitivity analysis at a minimum follow‐up of 12 months (MD = −0.92 [95% CI: −2.6 to 0.75], *p* = 0.28) (Supporting Information S1: Figure 2).

### Tegner activity score

**Figure 6:**
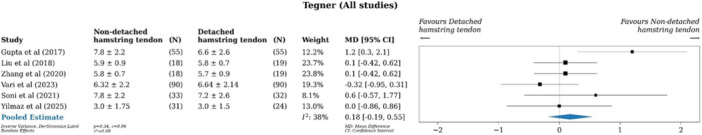
Meta‐analysis of studies reporting Tegner score at the latest follow‐up. CI, confidence interval; MD, mean difference.

A total of seven studies reported Tegner scores at various follow‐up periods [3, 20, 33, 40, 47, 50, 54, 56]. There was only one study that reported Tegner scores at 3‐month follow‐up, showing no significant difference between groups (DHT: 2.3 ± 0.7 vs. NDHT: 2.4 ± 0.7; *p* > 0.05) [20]. At 6 months, two studies found no significant difference [50, 56], while another study reported significantly higher scores in the NDHT group (*p* < 0.01), though exact values were not provided [3]. At 12 months, five studies found no significant differences between groups [3, 20, 33, 47, 56]. On meta‐analysis across included studies at the latest follow‐up of minimum of 6 months showed no significant difference between the two groups (MD = 0.18, [95% CI: −0.19 to 0.55], *p* = 0.34) (Figure 6). These findings did not change on sensitivity analysis at a minimum follow‐up of 24 months (MD = 0.35 [95% CI: −0.21 to 0.92], *p* = 0.22) (Supporting Information S1: Figure 3).

### Knee laxity arthrometer testing

**Figure 7:**
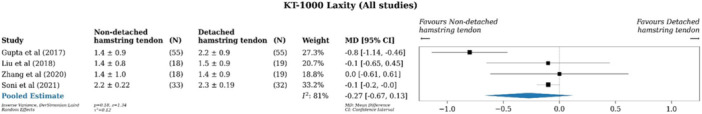
Meta‐analysis of studies reporting KT‐1000 Laxity scores at the latest follow‐up. CI, confidence interval; KT‐1000, knee testing 1000 (laxity arthrometer); MD, mean difference.

A total of five studies reported KT‐1000 arthrometer scores at various follow‐up intervals [12, 13, 20, 40, 56]. At 3 months, two studies found no significant difference between DHT and NDHT groups [13, 20]. At 6 months, three studies reported KT‐1000 scores [13, 20, 56]. Of these, only one study found a significant difference favouring the NDHT group (2.0 ± 1.2 vs. 2.8 ± 1.8; *p* < 0.01) [13], indicating reduced anterior laxity, while two studies found no difference [20, 56]. One study assessed KT‐1000 at 4 months and reported significant improvement from baseline only in the NDHT group (*p* < 0.01) [12]. However, this difference was no longer observed at 18 months (*p* > 0.05) [12]. At 12 months, three studies reported KT‐1000 scores, with one study showing a significant difference (NDHT: 1.5 ± 0.9 vs. DHT: 2.5 ± 1.5; *p* < 0.01) [13], while the two other studies reported no significant differences [13, 20, 56]. At ≥24 months, four studies were included in the meta‐analysis, which showed no significant difference in KT‐1000 scores between groups (MD = −0.29 [95% CI: −0.67 to 0.30]; *p* = 0.14; Figure 7) [13, 20, 40, 56]. This finding did not change on sensitivity analysis at a minimum follow‐up of 24 months (MD = −0.35 [95% CI: −0.9 to 0.21], *p* = 0.23) (Supporting Information S1: Figure 4).

### RTS and retear rates

**Figure 8:**
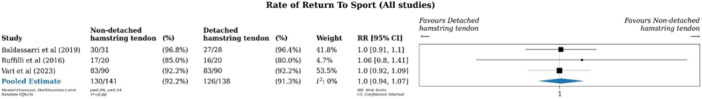
Meta‐analysis of studies reporting rate of return‐to‐sport at the latest follow‐up. CI, confidence interval; RR, risk ratio.

A total of three studies reported rates of RTS at ≥12‐month follow‐up, all of which were included in the meta‐analysis [3, 33, 47]. Pooled analysis at minimum follow‐up of 12 months showed no significant difference between DHT and NDHT groups (RR = 1.00 [95% CI: 0.94–1.07]; *p* = 0.89; Figure 8). These findings did not change on sensitivity analysis at minimum follow‐up of 24 months (MD = 1.01 [95% CI: 0.92 to 1.11], *p* = 0.84) (Supporting Information S1: Figure 5).

**Figure 9:**
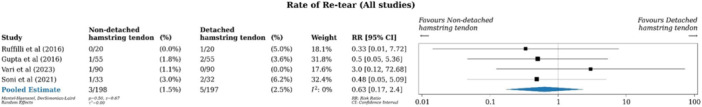
Meta‐analysis of included studies reporting rate of re‐tear at the latest follow‐up. CI, confidence interval; RR, risk ratio.

Similarly, four studies reported retear rates at ≥12 months and were included in the meta‐analysis [13, 33, 40, 47]. Although the NDHT group demonstrated a 63% lower risk of retear compared to DHT, this did not reach statistical significance (RR = 0.63 [95% CI: 0.17–2.40]; *p* = 0.50; Figure 9) [13, 33, 40, 47]. These findings did not change the sensitivity of analysis at minimum follow‐up of 24 months (MD = 0.43, [95% CI: 0.07 to 2.87], *p* = 0.38) (Supporting Information S1: Figure 6).

### Graft maturation

A total of four assessed graft maturation [10, 20, 47, 56]. Of these, three evaluated the graft maturation in tibial tunnel using SNQ [20, 47, 56], and one study used Figueroa scores [10].

Importantly, the ACL graft from the entire intra‐articular portion was measured, but the SNQ was calculated differently [20, 47, 56]. Specifically, one study used a single middle sagittal slice divided into proximal, mid‐substance and distal thirds and averaging the values [20]. Another study used a transverse slice tangent to the ACL at three levels and averaging them [47]. Another study divided the graft into the femoral tunnel, intra‐articular (middle portion) and tibial tunnel regions, reporting each separately [56] (Table 2).

**Table 2:** SNQ and tibial tunnel widening reported in the included studies.

Study (Reference)	SNQ formula	Follow‐up	SNQ		*p* value
			NDHT	DHT	
			Mean (±SD)	Mean (±SD)	
Liu et al. (2018) [20]	(Signal of ACL graft − Signal of quadriceps tendon)/(Background signal)	3 months	15.0 ( ± 11.2)	21.4 ( ± 12.7)	*p* = 0.11
		6 months	14.9 ( ± 6.3)	25.6 ( ± 12)	*p* = 0.002
		12 months	12.6 ( ± 7.0)	18.3 ( ± 7.7)	*p* = 0.02
		24 months	14.6 ( ± 7.0)	15.3 ( ± 6.3)	*p* = 0.75
Zhang et al. (2020) [56]	(Signal of ACL graft)/(Background signal)	6 months	IAG: 11.9 ( ± 5.6)	IAG: 22.1 ( ± 8.7).	IAG: *p* < 0.001
			FTG: 22.9 (7.4)	FTG: 31.9 (12.2)	FTG: *p* = 0.002
			TTG: 10.5 (5.7)	TTG: 16.4 (6.2)	TTG: *p* < 0.001
		12 months	IAG: 10.7 ( ± 5.5)	IAG: 17.3 ( ± 6.4)	IAG: *p* = 0.002
			FTG: 17.0 (7.4)	FTG: 23.2 (7.8)	FTG: *p* = 0.001
			TTG: 9.4 (5.2)	TTG: 13.5 (5.5)	TTG: *p* = 0.002
		24 months	IAG: 10.1 ( ± 7.2)	IAG: 12.7 ( ± 6.9)	IAG: *p* = 0.27
			FTG: 11.1 (8.3)	FTG: 16.9 (8.2)	FTG: *p* = 0.007
			TTG: 10.8 (6.3)	TTG: 11.6 (5.8)	TTG: *p* = 0.61
		60 months	IAG: 10.3 ( ± 4.8)	IAG: 11.2 ( ± 5.7)	IAG: *p* = 0.63
			FTG: 11.0 (5.8)	FTG: 11.5 (5.5)	FTG: *p* = 0.68
			TTG: 10.2 (4.8)	TTG: 10.6 (5.3)	TTG: *p* = 0.67
Vari et al. (2023) [46]	(Signal of ACL graft − Signal of PCL graft)/(Background signal)	12 months	3.9 ( ± 2.9)	1.2 ( ± 1.0)	*p* < 0.001

Abbreviations: ACL, anterior cruciate ligament; DHT, detached hamstring tendon graft; FTG, femoral intratunnel graft region; IAG, intra‐articular graft region; NA, not applicable; NDHT, non‐detached hamstring tendon graft; NR, not reported; PCL, posterior cruciate ligament; SD, standard deviation; SNQ, signal‐to‐noise quotient; TTG, tibial intratunnel graft region.

One study calculated SNQ as (ACL graft signal − quadriceps tendon signal)/background signal [20]. Another used (ACL graft signal − posterior cruciate ligament [PCL] signal)/background signal [47], and one divided ACL graft signal directly by the background signal [56] (Table 2). Only one study reported SNQ at 3 months and found no significant difference between groups [20]. At 6 months, two studies demonstrated significantly lower SNQ values in the NDHT group compared to DHT (*p* < 0.01; Table 2) [20, 56]. At 12 months, all three studies showed significantly lower SNQ in the NDHT group (*p* < 0.05), indicating more advanced graft maturation and remodelling, with tissue characteristics closer to native ligament on MRI [20, 47, 56] (Table 2). By ≥24 months, two studies found no significant SNQ differences between groups (*p* > 0.05; Table 2) [20, 56].

One study showed superior graft maturation outcomes at 6 months using Figueroa scoring system, where NDHT group showed higher integration scores (1.62 ± 0.49 vs. 1.06 ± 0.23; *p* < 0.01), higher ligamentization scores (2.15 ± 0.53 vs. 1.28 ± 0.45; *p* < 0.01) and higher total Figueroa scores (3.77 ± 0.89 vs. 2.33 ± 0.47; *p* < 0.01) compared with the DHT group, and reported 2.78‐fold increased likelihood of adequate graft maturation in NDHT (*p* < 0.01) at 6 months [10].

## DISCUSSION

The primary finding of this review is that there are no clinically meaningful differences between NDHT and DHT ACLR with respect to patient‐reported outcomes (IKDC, Lysholm, Tegner), objective knee stability (KT‐1000), RTS or re‐tear rates at the latest follow‐up of minimum of 6 months. Although some individual studies reported early differences favouring NDHT at 3‐ and 6‐month follow‐up [3, 10, 13], these differences were not sustained over time and were not confirmed in pooled meta‐analyses. These findings did not change in the sensitivity analysis. Radiologically, NDHT grafts demonstrated faster maturation at 6 and 12 months compared to DHT grafts, but these differences were no longer evident at ≥24 months [20, 47, 56].

The early clinical advantages seen in some studies [3, 10, 13] may be attributed to the preservation of the hamstring graft's tibial insertion, which helps maintain its vascular network, potentially accelerating the ligamentization process [53]. This enhanced vascularity may also improve proprioceptive input, enabling more effective rehabilitation [7, 22, 29, 33, 51, 52]. However, these proposed mechanisms did not translate into sustained or clinically significant differences in pooled clinical outcomes.

These differences disappeared in meta‐analyses of at least 12 months. This may be due to completion of graft ligamentization and neuromuscular adaptation in both groups, leading to convergence in mechanical outcomes over time [12, 13, 51, 53].

One study showed that the time to RTS was significantly shorter in the NDHT group (249 ± 142 days) compared to the DHT group (317 ± 145 days) (*p* = 0.002) [47]. However, the pooled analysis presented in this study showed no significant difference in the *rate* of RTS between both groups. A plausible explanation is that the early biological and clinical advantages associated with NDHT may accelerate initial recovery, thereby shortening the time to return, but do not ultimately influence whether patients RTS. Over the long term, both techniques yield comparable functional outcomes, which might explain why time to return may differ while RTS rates remain similar [47].

MRI‐based findings align with this trend, as NDHT was associated with significantly lower SNQ values at early follow‐up intervals (6 and 12 months), reflecting accelerated and more advanced graft maturation in the NDHT group [20, 47, 56]. By 24 and 60 months, these differences disappeared, suggesting that graft maturation ultimately reaches a similar level in both groups over the long term [20, 47, 56]. Importantly, these findings may support a surgical preference towards NDHT in younger athletes who would benefit from a faster RTS, even if long‐term outcomes are expected to converge between both groups. However, based on the findings of this study, no clinically meaningful advantage was demonstrated for either technique with respect to RTS outcomes.

This accelerated graft remodelling seen with NDHT aligns with anatomical and histological evidence. One study described the pes anserinus tendons as having a robust neurovascular supply, with vessel calibre decreasing proximally from the tibial insertion, vascularity that is preserved in NDHT but lost in DHT [53]. Supporting this, another study conducted a rabbit model study comparing NDHT and DHT grafts at 3, 6 and 12 weeks postoperatively [28]. They observed that NDHT grafts showed no necrosis by Week 3, retained native cellularity and structure and resembled normal ligament tissue by Week 12 [28]. In contrast, DHT grafts demonstrated disorganized architecture and degenerative changes [28]. Despite these findings, the findings of the present study indicate no clinically meaningful differences in patient‐reported outcomes, knee stability or RTS rates between the two techniques.

Adopting the NDHT may enhance surgical efficiency. In the DHT technique, detaching the hamstring tendons requires additional steps such as more extensive graft harvesting, preparation and secure fixation of the free ends, which may prolong operative time. In contrast, NDHT preserves the tibial insertion, thereby eliminating these steps and potentially reducing intraoperative duration. Beyond the potential surgical efficiency, there may be meaningful economic implications as well. A cost‐effectiveness analysis would be valuable to evaluate the potential financial benefits of this approach. Specifically, preserving the tibial insertion in NDHT may reduce or eliminate the need for additional tibial fixation devices, which represent a significant component of procedural cost, as tibial fixation costs alone were reported to range from $95.00 to $760.00 USD, with a mean cost of $293.52 USD per case [1].

In addition, NDHT makes double fixation possible: native tendon fixation and supplemental mechanical interference screw fixation [2, 38]. This dual fixation may provide greater primary mechanical stability and may reduce the risk of graft slippage or early failure. This was supported by a cadaveric study, which showed that load to failure was 33% higher in the NDHT group (with preserved insertion and no screw) compared to the DHT group (detached graft fixed with a screw) [2]. When an interference screw was added in the NDHT group, failure strength increased by an additional 25%, resulting in a 65% higher pull‐out strength compared to standard single screw fixation in DHT [2]. Although these biomechanical advantages may suggest improved graft security, the findings of this study did not demonstrate any corresponding differences in clinical outcomes or patient‐reported measures between NDHT and DHT techniques.

To the authors' knowledge, this is the largest, most comprehensive and up‐to‐date systematic review and meta‐analysis. It is the first to compare clinical outcomes, including IKDC, Lysholm, RTS and KT‐1000 scores and to evaluate graft maturation across the included studies. Importantly, this work provides several key contributions to the existing NDHT literature. First, while a previous systematic review broadly compared NDHT to multiple ACL graft types [46], a specific focus on NDHT versus standard detached hamstring grafts was applied in the present analysis. In addition, a broader and more systematic search strategy ultimately identified six additional studies not included in the most recent review [3, 10, 37, 40, 47, 50]. Furthermore, that systematic review included studies with concomitant extra‐articular procedures, whereas the present study excluded these studies to ensure a methodologically accurate comparison. This is a significant confounding factor as an extra‐articular procedure is known to affect graft maturation and MRI signal [32]. Moreover, several analyses previously absent from the literature were introduced, including sensitivity analyses, pooled assessments of RTS and re‐tear rates and stratification of clinical and radiological outcomes by follow‐up period to better characterize early versus long‐term effects. Collectively, these additions provide the most detailed and methodologically robust evaluation to date of how tibial‐insertion preservation influences outcomes in hamstring‐based ACL reconstruction.

Despite the strengths of this review, it has some limitations. First, the included data were derived from surgeries performed by various surgeons, introducing some variability in studies that utilized different, though comparable, surgical techniques. In addition, this analysis did not distinguish between studies based on the use of double fixation or on whether both the ST and G tendons were preserved. Specifically, while the surgical techniques use the same hamstring tendons, there are differences between them, particularly in the double fixation method used (screw, Endobutton, staple), which can introduce bias in the results. In addition, one of the included studies included a double tunnel technique [29], which might affect the generalizability of the findings. However, this study was not included in any of the meta‐analyses. Lastly, the analysis was constrained by incomplete reporting of certain outcomes in some of the studies. Future high‐quality studies are needed to confirm these findings, evaluate long‐term clinical and economic impact and further guide surgical decision‐making.

## CONCLUSION

Preserving the tibial insertion of the hamstring graft in ACLR (NDHT) resulted in comparable clinical outcomes to those of the detached technique (DHT), with no significant differences observed between the two approaches. There may be early radiological benefits associated with NDHT. However, larger high‐quality randomized studies are needed to draw stronger conclusions. Given the high global volume of hamstring‐based ACLRs, future studies are needed to evaluate the potential impact of preserving the hamstring tibial insertion on overall cost savings and operative time.

## AUTHOR CONTRIBUTIONS

All authors contributed to study design, data collection, analysis and manuscript preparation.

## CONFLICT OF INTEREST STATEMENT

Darren de SA has the following disclosures, none of which are related to this publication. He is a board member of the Heron Therapeutics Advisory Board and has served as a consultant for L.E.K. Consulting, Atheneum Partners and Stryker. Additionally, he has participated in the Speakers Bureau for ConMed Linvatec and is a member of the Pendopharm Regional Working Group. Amit Meena reports consulting roles with DePuy Mitek Sports Medicine and Maxx Orthopedics, none of which are affiliated with this publication. The remaining authors declare no conflict of interest.

## ETHICS STATEMENT

The authors have nothing to report.

## Supporting information


Supporting File

## Data Availability

The data that support the findings of this study are available in the supporting information of this article.

## References

[1] Archibald‐Seiffer N , Jacobs Jr. JC , Saad C , Jevsevar DS , Shea KG . Review of anterior cruciate ligament reconstruction cost variance within a regional health care system. Am J Sports Med. 2015;43:1408–1412. 25899430 10.1177/0363546515579184

[2] Bahlau D , Clavert P , Favreau H , Ollivier M , Lustig S , Bonnomet F , et al. Mechanical advantage of preserving the hamstring tibial insertion for anterior cruciate ligament reconstruction—a cadaver study. Orthop Traumatol Surg Res. 2019;105:89–93. 30579723 10.1016/j.otsr.2018.11.014

[3] Baldassarri M , Perazzo L , Ghinelli D , Ricciarelli M , Pilla F , Buda R . Return to sport after ACL surgery: a comparison between two different reconstructive techniques. J Knee Surg. 2019;32:513–518. 29791924 10.1055/s-0038-1653948

[4] Bourke HE , Salmon LJ , Waller A , Patterson V , Pinczewski LA . Survival of the anterior cruciate ligament graft and the contralateral ACL at a minimum of 15 years. Am J Sports Med. 2012;40:1985–1992. 22869626 10.1177/0363546512454414

[5] Bram JT , Magee LC , Mehta NN , Patel NM , Ganley TJ . Anterior cruciate ligament injury incidence in adolescent athletes: a systematic review and meta‐analysis. Am J Sports Med. 2021;49:1962–1972. 33090889 10.1177/0363546520959619

[6] Buda R , Di Caprio F , Giuriati L , Luciani D , Busacca M , Giannini S . Partial ACL tears augmented with distally inserted hamstring tendons and over‐the‐top fixation: an MRI evaluation. Knee. 2008;15:111–116. 18262424 10.1016/j.knee.2007.12.002

[7] Buda R , Ruffilli A , Vannini F , Parma A , Giannini S . Anatomic anterior cruciate ligament reconstruction using distally inserted doubled hamstrings tendons. Orthopedics. 2013;36:449–453. 23746007 10.3928/01477447-20130523-04

[8] Claes S , Verdonk P , Forsyth R , Bellemans J . The “ligamentization” process in anterior cruciate ligament reconstruction: what happens to the human graft? A systematic review of the literature. Am J Sports Med. 2011;39:2476–2483. 21515806 10.1177/0363546511402662

[9] Crawford C , Nyland J , Landes S , Jackson R , Chang HC , Nawab A , et al. Anatomic double bundle ACL reconstruction: a literature review. Knee Surg Sports Traumatol Arthrosc. 2007;15:946–964; discussion 945. 17534599 10.1007/s00167-007-0343-7

[10] Espinoza Dumlao III P , Lovina Bathan L , Mia Dizon P . Preserved tibial attachment of hamstring graft versus detached graft in anterior cruciate ligament reconstruction: a randomized controlled study. J Surg. 2020;8:62–66.

[11] Grassi A , Carulli C , Innocenti M , Mosca M , Zaffagnini S , Bait C , et al. New trends in anterior cruciate ligament reconstruction: a systematic review of national surveys of the last 5 years. Joints. 2018;6:177–187. 30582107 10.1055/s-0038-1672157 PMC6301855

[12] Grassi A , Casali M , Macchiarola L , Lucidi GA , Cucurnia I , Filardo G , et al. Hamstring grafts for anterior cruciate ligament reconstruction show better magnetic resonance features when tibial insertion is preserved. Knee Surg Sports Traumatol Arthrosc. 2021;29:507–518. 32266415 10.1007/s00167-020-05948-z

[13] Gupta R , Bahadur R , Malhotra A , Masih GD , Sood M , Gupta P , et al. Outcome of hamstring autograft with preserved insertions compared with free hamstring autograft in anterior cruciate ligament surgery at 2‐year follow‐up. Arthroscopy. 2017;33:2208–2216. 28969952 10.1016/j.arthro.2017.06.040

[14] Higgins JPT , Altman DG , Gotzsche PC , Juni P , Moher D , Oxman AD , et al. The Cochrane Collaboration's tool for assessing risk of bias in randomised trials. BMJ. 2011;343:d5928. 22008217 10.1136/bmj.d5928 PMC3196245

[15] Hozo SP , Djulbegovic B , Hozo I . Estimating the mean and variance from the median, range, and the size of a sample. BMC Med Res Methodol. 2005;5:13. 15840177 10.1186/1471-2288-5-13 PMC1097734

[16] Hui C , Salmon LJ , Kok A , Maeno S , Linklater J , Pinczewski LA . Fifteen‐year outcome of endoscopic anterior cruciate ligament reconstruction with patellar tendon autograft for “isolated” anterior cruciate ligament tear. Am J Sports Med. 2011;39:89–98. 20962336 10.1177/0363546510379975

[17] Kaeding CC , Pedroza AD , Reinke EK , Huston LJ , Spindler KP , Amendola A , et al. Risk factors and predictors of subsequent ACL injury in either knee after ACL reconstruction: prospective analysis of 2488 primary ACL reconstructions from the MOON cohort. Am J Sports Med. 2015;43:1583–1590. 25899429 10.1177/0363546515578836 PMC4601557

[18] Kim SJ , Kim HK , Lee YT . Arthroscopic anterior cruciate ligament reconstruction using autogenous hamstring tendon graft without detachment of the tibial insertion. Arthroscopy. 1997;13:656–660. 9343660 10.1016/s0749-8063(97)90198-5

[19] Lai VJ , Reynolds AW , Kindya M , Konicek J , Akhavan S . The use of suture augmentation for graft protection in ACL reconstruction: a biomechanical study in porcine knees. Arthrosc Sports Med Rehabil. 2021;3:e57–e63. 33615248 10.1016/j.asmr.2020.08.009 PMC7879175

[20] Liu S , Li H , Tao H , Sun Y , Chen S , Chen J . A randomized clinical trial to evaluate attached hamstring anterior cruciate ligament graft maturity with magnetic resonance imaging. Am J Sports Med. 2018;46:1143–1149. 29443537 10.1177/0363546517752918

[21] Marcacci M , Molgora AP , Zaffagnini S , Vascellari A , Iacono F , Presti ML . Anatomic double‐bundle anterior cruciate ligament reconstruction with hamstrings. Arthroscopy. 2003;19:540–546. 12724685 10.1053/jars.2003.50129

[22] Marcacci M , Zaffagnini S , Giordano G , Iacono F , Lo Presti M . Anterior cruciate ligament reconstruction associated with extra‐articular tenodesis: a prospective clinical and radiographic evaluation with 10‐ to 13‐year follow‐up. Am J Sports Med. 2009;37:707–714. 19193599 10.1177/0363546508328114

[23] Marks Benson E , Pyrz K , Wood A , Momaya A , Brabston E , Evely T , et al. Anterior cruciate ligament reconstruction using bone‐tendon‐bone allograft: surgical technique using augmentation with bio‐composite scaffold. Arthrosc Tech. 2024;13:102877. 38584643 10.1016/j.eats.2023.11.005 PMC10995643

[24] Mascarenhas R , Erickson BJ , Sayegh ET , Verma NN , Cole BJ , Bush‐Joseph C , et al. Is there a higher failure rate of allografts compared with autografts in anterior cruciate ligament reconstruction: a systematic review of overlapping meta‐analyses. Arthroscopy. 2015;31:364–372. 25220350 10.1016/j.arthro.2014.07.011

[25] Montalvo AM , Schneider DK , Yut L , Webster KE , Beynnon B , Kocher MS , et al. “What's my risk of sustaining an ACL injury while playing sports?” A systematic review with meta‐analysis. Br J Sports Med. 2019;53:1003–1012. 29514822 10.1136/bjsports-2016-096274 PMC6561829

[26] Mouarbes D , Menetrey J , Marot V , Courtot L , Berard E , Cavaignac E . Anterior cruciate ligament reconstruction: a systematic review and meta‐analysis of outcomes for quadriceps tendon autograft versus bone‐patellar tendon‐bone and hamstring‐tendon autografts. Am J Sports Med. 2019;47:3531–3540. 30790526 10.1177/0363546518825340

[27] Page MJ , McKenzie JE , Bossuyt PM , Boutron I , Hoffmann TC , Mulrow CD , et al. The PRISMA 2020 statement: an updated guideline for reporting systematic reviews. BMJ. 2021;372:n71. 33782057 10.1136/bmj.n71 PMC8005924

[28] Papachristou G , Nikolaou V , Efstathopoulos N , Sourlas J , Lazarettos J , Frangia K , et al. ACL reconstruction with semitendinosus tendon autograft without detachment of its tibial insertion: a histologic study in a rabbit model. Knee Surg Sports Traumatol Arthrosc. 2007;15:1175–1180. 17622515 10.1007/s00167-007-0374-0

[29] Papachristou G , Sourlas J , Plessas S , Papachristou K . Arthroscopic ACL reconstruction with Δ plasty: an innovative approach with hamstrings' transfer and double tibial tunnel. Knee Surg Sports Traumatol Arthrosc. 2008;16:420–426. 17934715 10.1007/s00167-007-0426-5

[30] Pauzenberger L , Syré S , Schurz M . “Ligamentization” in hamstring tendon grafts after anterior cruciate ligament reconstruction: a systematic review of the literature and a glimpse into the future. Arthroscopy. 2013;29:1712–1721. 23859954 10.1016/j.arthro.2013.05.009

[31] Roger J , Bertani A , Vigouroux F , Mottier F , Gaillard R , Have L , et al. ACL reconstruction using a quadruple semitendinosus graft with cortical fixations gives suitable isokinetic and clinical outcomes after 2 years. Knee Surg Sports Traumatol Arthrosc. 2020;28:2468–2477. 32699919 10.1007/s00167-020-06121-2

[32] Rojas G , Perelli S , Ibanez M , Formagnana M , Ormazabal I , Monllau JC . Effect of modified Lemaire anterolateral extra‐articular tenodesis on the magnetic resonance imaging maturity signal of anterior cruciate ligament hamstring graft. Am J Sports Med. 2021;49:2379–2386. 34133234 10.1177/03635465211018858

[33] Ruffilli A , Pagliazzi G , Ferranti E , Busacca M , Capannelli D , Buda R . Hamstring graft tibial insertion preservation versus detachment in anterior cruciate ligament reconstruction: a prospective randomized comparative study. Eur J Orthop Surg Traumatol. 2016;26:657–664. 27388213 10.1007/s00590-016-1812-9

[34] Samuelsen BT , Webster KE , Johnson NR , Hewett TE , Krych AJ . Hamstring autograft versus patellar tendon autograft for ACL reconstruction: is there a difference in graft failure rate? A meta‐analysis of 47,613 patients. Clin Orthop Relat Res. 2017;475:2459–2468. 28205075 10.1007/s11999-017-5278-9 PMC5599382

[35] Sanders TL , Maradit Kremers H , Bryan AJ , Larson DR , Dahm DL , Levy BA , et al. Incidence of anterior cruciate ligament tears and reconstruction: a 21‐year population‐based study. Am J Sports Med. 2016;44:1502–1507. 26920430 10.1177/0363546516629944

[36] Silva A , Sampaio R . Anatomic ACL reconstruction: does the platelet‐rich plasma accelerate tendon healing? Knee Surg Sports Traumatol Arthrosc. 2009;17:676–682. 19288080 10.1007/s00167-009-0762-8

[37] Singh A , Agrawal A , Khera R , Siddiqi AK . Functional outcome of anterior cruciate ligament reconstruction by tibial attachment preserving versus sacrificing hamstring graft technique—a prospective interventional study. J Clin Diagn Res. 2022;16:10–14.

[38] Sinha S , Naik AK , Maheshwari M , Sandanshiv S , Meena D , Arya RK . Anterior cruciate ligament reconstruction with tibial attachment preserving hamstring graft without implant on tibial side. Indian J Orthop. 2018;52:170–176. 29576645 10.4103/ortho.IJOrtho_85_17 PMC5858211

[39] Slim K , Nini E , Forestier D , Kwiatkowski F , Panis Y , Chipponi J . Methodological index for non‐randomized studies (minors): development and validation of a new instrument. ANZ J Surg. 2003;73:712–716. 12956787 10.1046/j.1445-2197.2003.02748.x

[40] Soni A , Gupta RK , Raghav M , Masih GD , Bansal P . Comparison of bone‐patellar tendon‐bone graft, semitendinosus‐gracilis graft and semitendinosus‐gracilis with preserved tibial insertion graft in anterior cruciate ligament reconstruction in sports persons. Malays Orthop J. 2021;15:12–17. 10.5704/MOJ.2107.003 PMC8381676 34429817

[41] Sonnery‐Cottet B , Freychet B , Murphy CG , Pupim BH , Thaunat M . Anterior cruciate ligament reconstruction and preservation: the single‐anteromedial bundle biological augmentation (SAMBBA) technique. Arthrosc Tech. 2014;3:e689–e693. 25685675 10.1016/j.eats.2014.08.007 PMC4314548

[42] Sterne JA , Hernán MA , Reeves BC , Savović J , Berkman ND , Viswanathan M , et al. ROBINS‐I: a tool for assessing risk of bias in non‐randomised studies of interventions. BMJ. 2016;355:i4919. 27733354 10.1136/bmj.i4919 PMC5062054

[43] Sun B , Lee B , Grad J , Cohen D , Abouali J , Tapasvi S , et al. Anterior cruciate ligament reconstruction with six and eight‐strand hamstring tendon autografts produces adequate graft dimensions and functional outcomes: a systematic review. Knee Surg Sports Traumatol Arthrosc. 2025;33:2144–2155. 39666599 10.1002/ksa.12556 PMC12104794

[44] Tuca M , Valderrama I , Eriksson K , Tapasvi S . Current trends in anterior cruciate ligament surgery. A worldwide benchmark study. J ISAKOS. 2023;8:2–10. 36154898 10.1016/j.jisako.2022.08.009

[45] van Eck CF , Schkrohowsky JG , Working ZM , Irrgang JJ , Fu FH . Prospective analysis of failure rate and predictors of failure after anatomic anterior cruciate ligament reconstruction with allograft. Am J Sports Med. 2012;40:800–807. 22238055 10.1177/0363546511432545

[46] Vari N , Cavaignac E , Cavaignac M , Bérard É , Marot V . Outcomes of hamstring graft with preserved tibial insertion for ACL reconstruction: systematic review and meta‐analysis. Eur J Orthop Surg Traumatol. 2024;34:67–73. 37644333 10.1007/s00590-023-03698-5 PMC10771374

[47] Vari N , Marot V , Ripoll T , Vieira TD , Martinel V , Bérard E , et al. Preserving the semitendinosus distal attachment is associated with improved graft remodeling after ACL reconstruction. Am J Sports Med. 2023;51:2064–2072. 37204156 10.1177/03635465231169047

[48] Wicks ED , Stack J , Rezaie N , Zeini IM , Osbahr DC . Biomechanical evaluation of suture tape internal brace reinforcement of soft tissue allografts for ACL reconstruction using a porcine model. Orthop J Sports Med. 2022;10:23259671221091252. 35547611 10.1177/23259671221091252 PMC9083057

[49] Xie H , Fu Z , Zhong M , Deng Z , Wang C , Sun Y , et al. Effects of remnant preservation in anterior cruciate ligament reconstruction: a systematic review and meta‐analysis. Front Surg. 2022;9:952930. 36117844 10.3389/fsurg.2022.952930 PMC9475141

[50] Yilmaz M . The new auto graft technique in anterior cruciate ligament reconstruction. North Clin Istanb. 2025;12:1–11. 40838235 10.14744/nci.2023.40799 PMC12364475

[51] Zaffagnini S , Bruni D , Marcheggiani Muccioli GM , Bonanzinga T , Lopomo N , Bignozzi S , et al. Single‐bundle patellar tendon versus non‐anatomical double‐bundle hamstrings ACL reconstruction: a prospective randomized study at 8‐year minimum follow‐up. Knee Surg Sports Traumatol Arthrosc. 2011;19:390–397. 20668835 10.1007/s00167-010-1225-y

[52] Zaffagnini S , Bruni D , Russo A , Takazawa Y , Lo Presti M , Giordano G , et al. ST/G ACL reconstruction: double strand plus extra‐articular sling vs double bundle, randomized study at 3‐year follow‐up. Scand J Med Sci Sports. 2008;18:573–581. 18208432 10.1111/j.1600-0838.2007.00697.x

[53] Zaffagnini S , Golanò P , Farinas O , Depasquale V , Strocchi R , Cortecchia S , et al. Vascularity and neuroreceptors of the pes anserinus: anatomic study. Clin Anat. 2003;16:19–24. 12486734 10.1002/ca.10073

[54] Zaffagnini S , Marcacci M , Lo Presti M , Giordano G , Iacono F , Neri MP . Prospective and randomized evaluation of ACL reconstruction with three techniques: a clinical and radiographic evaluation at 5 years follow‐up. Knee Surg Sports Traumatol Arthrosc. 2006;14:1060–1069. 16909301 10.1007/s00167-006-0130-x

[55] Zaffagnini S , Marcheggiani Muccioli GM , Bonanzinga T , Nitri M , Grassi A , Marcacci M . Anatomic double‐bundle anterior cruciate ligament reconstruction leaving hamstrings tibial insertion intact: technical note. Musculoskelet Surg. 2013;97:39–43. 10.1007/s12306-012-0230-3 23233345

[56] Zhang Y , Liu S , Chen Q , Hu Y , Sun Y , Chen J . Maturity progression of the entire anterior cruciate ligament graft of insertion‐preserved hamstring tendons by 5 years: a prospective randomized controlled study based on magnetic resonance imaging evaluation. Am J Sports Med. 2020;48:2970–2977. 32909826 10.1177/0363546520951507

