# Supplementary material for: Non‐detached hamstring tendon anterior cruciate ligament reconstruction demonstrates comparable outcomes to the traditional detached hamstring tendon technique: A systematic review and meta‐analysis
Source: J Exp Orthop. 2026 May 5;13(2):e70705. doi: 10.1002/jeo2.70705 (PMC13140280; doi:10.1002/jeo2.70705)
Supplement: Supplementary file 1 — Supporting File [file JEO2-13-e70705-s001.docx]

**Supplementary Table i).** MeSH terms and Boolean operators used to develop search strategy from PubMed, Embase, OVID Medline, and CINAHL.

*Database: Embase <1974 to 2025 October 28>*

1. exp Anterior Cruciate Ligament/. Results: 16299
2. exp anterior cruciate ligament reconstruction/ or exp ligament surgery/. Results: 29569
3. Tibial insertion.mp. Results: 613
4. distal attachment.mp Results: 488
5. 2 and 5. Results: 18
6. 2 and 3. Reults: 217

*Database: OVID Medline <1964 to 2025 October 28>*

1. exp Anterior Cruciate Ligament/. Results: 12975
2. *Anterior Cruciate Ligament Reconstruction/. Results: 7476
3. Tibial insertion.mp. Results: 517
4. Distal Attachment.mp. Results: 295
5. Pedicle Preservation.mp. Results: 13
6. 2 and 5. Results: 2
7. 2 and 3. Results: 49

*Database: CINAHIL <1961 to 2025 October 28>*

1. S1 (MH "Surgery, Operative+") OR (MM "Anterior Cruciate Ligament Reconstruction"). Results: 785,419
2. S2 (MM "Anterior Cruciate Ligament Reconstruction"). Results: 6,763
3. S3 "Tibial insertion". Results: 192
4. S4 ("Tibial insertion") AND (S2 AND S3). Results: 43
5. S5 (MM "Anterior Cruciate Ligament Reconstruction") AND tibial preservation. Results: 0
6. S6 (("tibial preservation AND tibial preservation" AND (S3)). Results: 76

*Database: PubMed <1946 to 2025 October 28>*

("anterior cruciate ligament reconstruction"[MeSH] OR "ACL reconstruction" OR "ACLR" OR "anterior cruciate ligament surgery" OR "ACL surgery") AND ("tibial insertion" OR "tibial footprint" OR "distal insertion" OR "distal attachment" OR "insertion preservation" OR "tendon preservation" OR "pedicle preservation" OR "preserved tibial insertion" OR "remnant preservation" OR "remnant attachment" OR "tibial detachment" OR "detached graft" OR "graft detachment").


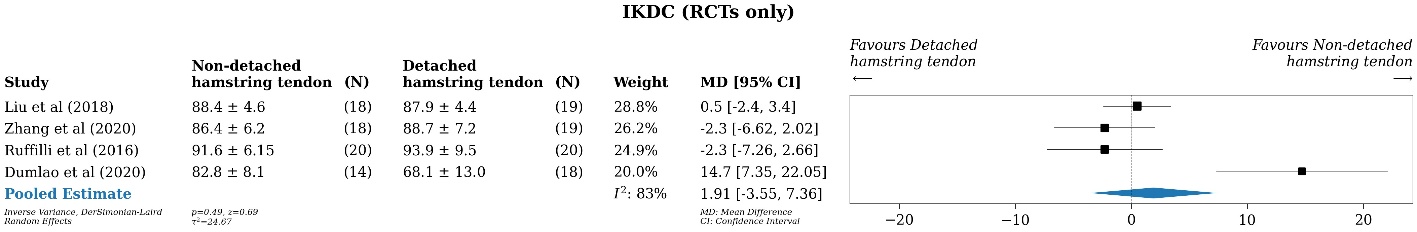


**Supplemental Figure S2.** Meta-analysis of IKDC across randomized controlled trials at latest follow-up.


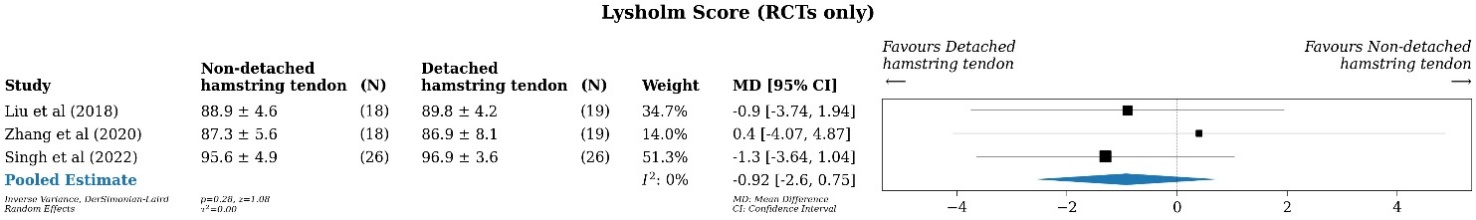


Supplemental Figure S3. Meta-analysis of Lysholm scores across randomized controlled trials at latest follow-up.


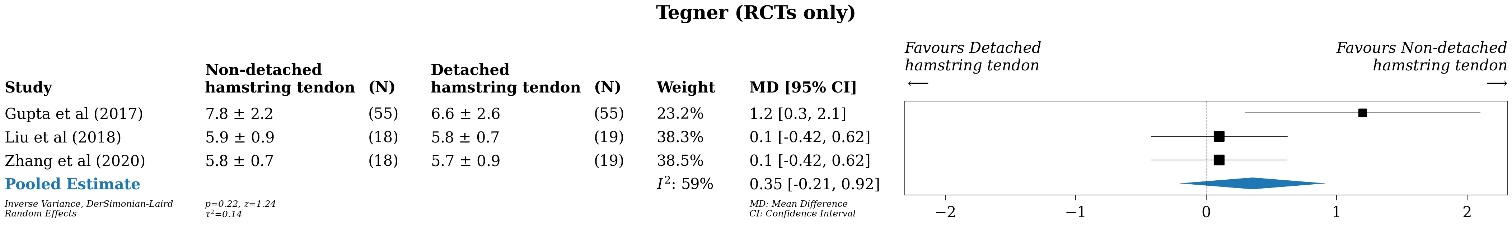


**Supplemental Figure S4**. Meta-analysis of Lysholm scores across randomized controlled trials at latest follow-up.


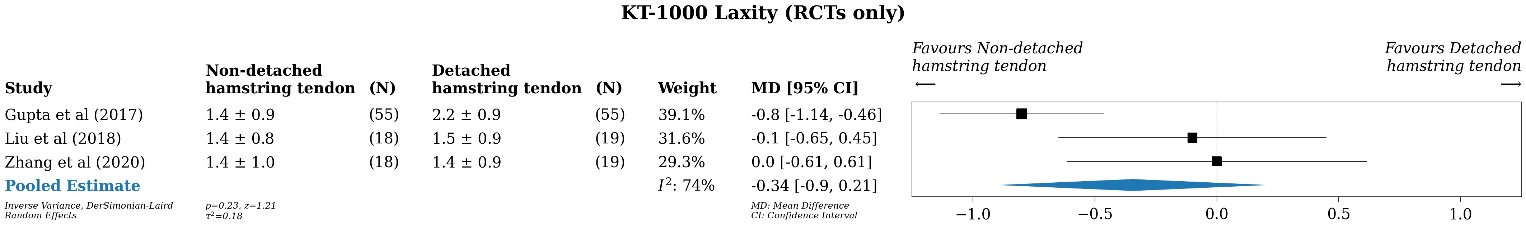


**Supplemental Figure S5**. Meta-analysis of Knee Laxity-1000 scores across randomized controlled trials at latest follow-up.


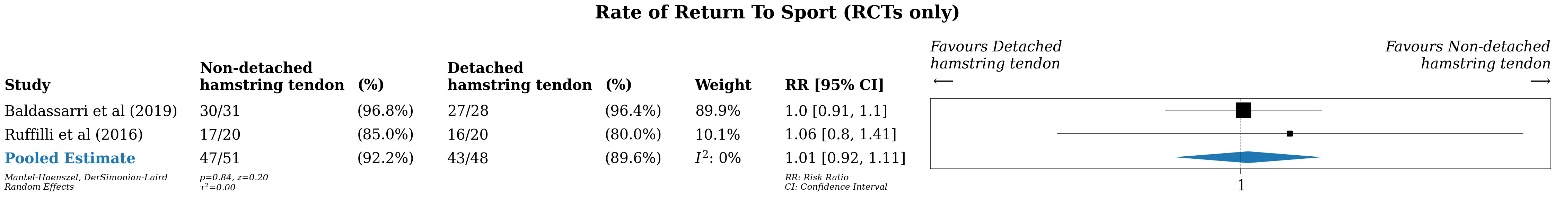


**Supplemental Figure S6**. Meta-analysis of return-to-sports across randomized controlled trials at latest follow-up.


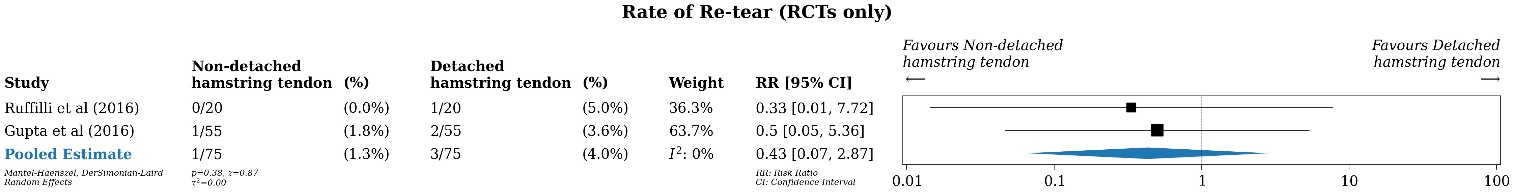


**Supplemental Figure S7**. Meta-analysis of rate of retear across randomized controlled trials at latest follow-up.
